# Association of Long-term Exposure to Air Pollution and Dementia Risk: The Role of Homocysteine, Methionine, and Cardiovascular Burden

**DOI:** 10.1212/WNL.0000000000207656

**Published:** 2023-09-19

**Authors:** Giulia Grande, Babak Hooshmand, Davide Liborio Vetrano, David A Smith, Helga Refsum, Laura Fratiglioni, Petter Ljungman, Jing Wu, Andrea Bellavia, Kristina Eneroth, Tom Bellander, Debora Rizzuto

**Affiliations:** From the Aging Research Center (G.G., B.H., D.L.V., L.F., J.W., D.R.), Department of Neurobiology, Care Sciences and Society, Karolinska Institutet and Stockholm University, Sweden; Department of Clinical Geriatrics (B.H.), Klinikum Ingolstadt, Germany; Stockholm Gerontology Research Centre (D.L.V., L.F., D.R.), Sweden; OPTIMA (D.S., H.R.), Department of Pharmacology, University of Oxford, United Kingdom; Department of Nutrition (H.R.), Institute of Basic Medical Sciences University of Oslo, Norway; Institute of Environmental Medicine (IMM) (P.L., T.B.), Karolinska Institutet; Department of Cardiology (P.L.), Danderyd Hospital, Stockholm, Sweden; Department of Environmental Health (A.B.), Harvard T.H. Chan School of Public Health, Boston, MA; and Environment and Health Administration (K.E.), City of Stockholm, Sweden.

## Abstract

**Background and Objectives:**

Growing evidence links air pollution with dementia risk, but the biological mechanisms are largely unknown. We investigated the role played by homocysteine (tHcy) and methionine in this association and explored whether this could be explained by cardiovascular diseases (CVDs).

**Methods:**

Data were extracted from the ongoing Swedish National study on Aging and Care in Kungsholmen (SNAC-K), a longitudinal population-based study. At baseline, 2,512 dementia-free participants were examined up to 2013 (mean follow-up: 5.18 ± 2.96 years). Two air pollutants (particulate matter ≤2.5 μm [PM_2.5_] and nitrogen oxides [NO_x_]) were assessed yearly from 1990 until 2013 using dispersion models at residential addresses. The hazard ratio of dementia over air pollution levels was estimated using Cox models adjusted for age, sex, education, smoking, socioeconomic status, physical activity, retirement age, creatinine, year of assessment, and the use of supplements. The total effect of air pollutants on dementia was decomposed into 4 pathways involving tHcy/methionine: (1) direct effect; (2) indirect effect (mediation); (3) effect due to interaction; and (4) effect due to both mediation and interaction. To test whether the association was independent from CVDs (ischemic heart disease, atrial fibrillation, heart failure, and stroke), we repeated the analyses excluding those individuals who developed CVDs.

**Results:**

The mean age of the study participants was 73.4 years (SD: 10.4), and 62.1% were female individuals. During an average period of 5 years (mean: 5.18; SD: 2.96 years), 376 cases with incident dementia were identified. There was a 70% increased hazard of dementia per unit increase of PM_2.5_ during the 5 years before baseline (hazard ratio [HR]: 1.71; 95% CI 1.33–2.09). Overall, 50% (51.6%; 95% CI 9.0–94.1) of the total effect of PM_2.5_ on dementia was due to mediation of tHcy (6.6%; 95% CI 1.6–11.6) and/or interaction (47.8%; 95% CI 4.9–91.7) with tHcy and 48.4% (*p* = 0.03) to the direct effect of PM_2.5_ on dementia. High levels of methionine reduced the dementia hazard linked to PM_2.5_ by 31% (HR: 0.69; 95% CI 0.56–0.85) with 24.8% attributable to the interaction with methionine and 25.9% (*p* = 0.001) to the direct effect of PM_2.5_. No mediation effect was found through methionine. Attenuated results were obtained for NO_x_. Findings for tHcy were attenuated after excluding those who developed CVDs, while remained similar for methionine.

**Discussion:**

High levels of homocysteine enhanced the dementia risk attributed to air pollution, while high methionine concentrations reduced this risk. The impact of homocysteine on cardiovascular conditions partly explains this association. Alternative pathways other than cardiovascular mechanisms may be at play between methionine and dementia.

## Introduction

A growing body of evidence has linked air pollution to negative cognitive outcomes, including dementia.^1-4^ Because air pollution is universal, this observation is of paramount importance because, even with small-to-moderate effect sizes, actions that aim to reduce air pollution would have an enormous public health impact in terms of dementia prevention.

Although most of the studies indicate an increased dementia risk linked to ambient air pollution, the mechanisms through which air pollution affects the brain are poorly understood. Findings from animal^5^ and human^6^ studies support the hypothesis that being exposed to polluted air can result in higher brain amyloid-β (Aβ) deposition and neurodegeneration. In addition, stroke is deemed as a relevant intermediate condition between air pollution and dementia, suggesting that vascular pathologies may also play a role in this relationship.^7,8^ Oxidative stress, endothelial dysfunction, and systemic inflammation have all been implicated in the pathogenesis of dementia and have been involved in both Aβ deposition and vascular damage.^9^ Homocysteine (tHcy), an amino acid generated through demethylation of methionine, may be an important contributor to these pathologic processes,^10^ and it has been linked to the development of cardiovascular diseases^10,11^ and dementia.^12,13^ Conversely, high levels of methionine, an essential amino acid and a precursor of tHcy, are associated with a decreased risk of cardiovascular and neurologic conditions including dementia.^14-16^ Few studies have explored the impact of air pollution on these amino acids, and a recent study found a positive association between high levels of particulate matter (PM) and tHcy and contrasting findings for methionine.^17^

In this study, we aimed to explore the impact of tHcy and methionine on the pathway linking air pollution to dementia. Furthermore, we examined whether cardiovascular diseases (CVDs) could have a role in explaining such association. We based our analyses on a clinically characterized population-based cohort with spatially highly resolved information on air pollution and longitudinal clinical evaluations of dementia.

## Methods

### Study Population

For this study, we used data from the ongoing *Swedish National study on Aging and Care in Kungsholmen* (SNAC-K), a population-based longitudinal study^18^ including individuals older than 60 years and residents of the Kungsholmen district in central Stockholm. At baseline (2001–2004), 3,363 (response rate 73.3%) participants were evaluated. Participants have then been followed up every 6 (young to old cohorts; aged 60–78 years) or 3 years (older cohorts; 78 years or older).

### Standard Protocol Approvals, Registrations, and Patient Consents

All participants or a proxy provided a written informed consent. The Regional Ethical Review Board in Stockholm, Sweden, approved the protocols of the SNAC-K study. This study was reported in keeping with the STROBE Recommendations (eTable 1, links.lww.com/WNL/C980).

### Data Collection

At each study visit, data were collected at a dedicated research center following standard procedures that included face-to-face interviews and clinical and laboratory examinations performed by trained physicians, nurses, and psychologists. Participants were assessed at home or in institution if they agreed to participate but were unable to reach the research center.

Information on age, sex, education, and retirement age were collected during the nurse interview. The highest level of formal education was categorized as elementary school, high school, and college/university or above. Socioeconomic position was operationalized considering the longest occupation held and was categorized into 3 groups^19^: blue and white collar workers and entrepreneurs. Retirement that occurred before the age of 65 years was considered as early retirement. Smoking was considered as current, former, or never smoker. Use of any type of vitamin B supplement was also collected during the physician interview, coupled with the drug register. Creatinine level was obtained from laboratory tests at baseline. The level of engagement in physical activities was derived from a questionnaire assessing both the frequency and intensity of different activities, and physical inactivity was defined whether the participant was active for less than once/week in light/intensive activity. DNA was extracted from venous blood and Apolipoprotein E (APOE) alleles genotyped. Participants have been categorized as ε4 and ε4 noncarriers.

For a subsample of SNAC-K participants (N = 1976), a self-administered semiquantitative questionnaire assessed food frequency and was used to retrieve dietary habits over the year before the assessment. The national food composition database was used to compute nutrient intake by multiplying each dish portion by the expected nutrient standard content.^20^

### Dementia Diagnosis

The clinical diagnosis of dementia was performed in keeping with the criteria of Diagnostic and Statistical Manual of Mental Disorders, Fourth Edition, based on the clinical examination conducted by physicians. In brief, it includes medical—with questions regarding comorbidities—and drug history and general and neurologic physical examination. Physicians assess cognitive functioning also by administering tests related to subjective cognitive complaints, problem-solving, abstract thinking, self-orientation and time-space orientation, and general knowledge. In addition, the Mini Mental State Examination (MMSE), Clock Drawing test, counting forward and backward, and a short story assessing frontal lobe are administered. Finally, independence of daily living both basic and instrumental are assessed. The diagnosis follows a procedure consisting in 3 steps.^21^ A first and preliminary diagnosis was made by the examining physician who met the participant; second, a second preliminary diagnosis was made by a reviewing physician from the data collection team. In case of disagreement between the first and the second diagnoses, the final diagnosis was made by senior neurologists not involved in the data collection. To further ascertain possible diagnoses of dementia among individuals who died between the SNAC-K follow-up examinations, clinical charts of those who died were collected with their death certificates and examined by the same physicians.

### Cardiovascular Disease Burden

We considered the following conditions as CVDs: ischemic heart disease, heart failure, atrial fibrillation, and stroke.

A comprehensive clinical procedure was followed to detect all diseases, as detailed elsewhere.^22^ In brief, diagnoses were based on medical history collected by physicians during the interviews, clinical examinations, diagnostic tests (instrumental and blood tests), inpatient and outpatient records, medical journals, and registers from the Swedish National Patient Register.^22^ Diagnoses were coded in accordance with the International Classification of Diseases, 10th revision on a clinical review performed by trained physicians.

### Air Pollution Assessment

We calculated annual mean levels of PM_2.5_ and NO_x_ at the home addresses of the participants with dispersion modeling according to local emission inventories.^23^ The inventories consist of local emissions of traffic and nontraffic sources for the following years: 1990, 1995, 2000, 2005, and 2011. The local emission of NO_x_ mainly consisted of exhaust emissions from road traffic, while residential wood burning, road traffic exhaust, and particles from road wear were the dominant sources of PM_2.5_. A Gaussian dispersion model was applied to the local emission databases. Annual mean levels of PM_2.5_ and NO_x_ were obtained from linear interpolation over the 4 years between each model simulation. To obtain total levels of PM_2.5_ and NO_x_, annual long-range contributions, homogeneous over the model domain, were added to the simulated locally generated levels. The long-range contributions were based on measurements at the rural monitoring site Norr Malma, located outside the calculation domain, 60 km northeast of Stockholm. We compared the model that calculated yearly levels with the one that measured the values at 3 curbside (traffic) monitoring sites and 1 urban background site in Stockholm City, and we obtained r^2^ values of 0.97 for NO_x_ and 0.86 for PM_2.5_.

Of the 2,512 participants included at baseline, 248 moved outside the district of Kungsholmen during the entire follow-up time. The exposure level of the pollutants for these individuals was calculated at the new home address. No major differences were detected between those who were residents of the Kungsholmen district for the entire period and those who moved outside the study area (5-year average before baseline of PM_2.5_ and NO_x_ were 8.4 ± 0.7 and 8.3 ± 0.7 and 33.9 ± 11.7 and 32.7 ± 11.7 for Kungsholmen residents and those who moved, respectively). Those who moved outside of Stockholm County (n = 9) were instead excluded from the study.

### Serum Methionine and Homocysteine

At baseline, nonfasting venous blood samples were collected. Routine analyses were conducted within 2 hours, through a chemiluminescence microparticle folate-binding protein assay at the Sabbatsberg Hospital, Stockholm, Sweden. Plates in dry ice were then shipped to the University of Oxford, United Kingdom. The levels of tHcy and methionine were measured using tandem mass spectrometry after treatment of the serum with a reducing agent, as previously detailed.^24^ Interassay coefficients of variation ranged between 5% and 10%. tHCy and methionine values above 15 µmol/L^25^ and 20.7 µmol/L (upper 2 tertiles) were considered as high, respectively. We also considered the ratio between methionine and tHcy (Met:tHcy) as a possible indicator of methylation activity with high ratios considered proxy for greater methylation activity.^15^ The 2 upper tertiles of Met:tHcy levels were defined as a high level (cutoff: 1.47 µmol/L).

### Statistical Analyses

Cox models were used to derive hazard ratios (HRs) and 95% confidence intervals (CIs) of dementia in relation to 5-year average PM_2.5_ and NO_x_ before baseline assessment. Individuals were considered at risk until dementia diagnosis, death, or end of follow-up, whichever came first. We assumed a linear relationship between PM_2.5_, NO_x_, and the log(HR). The proportional hazard assumption was assessed by regressing the scaled Schoenfeld residuals against survival time. No deviation from proportionality was detected (p: 0.3313 for PM_2.5_ and p: 0.1649 for NO_x_).

The potential mediating and interactive effects of serum markers of methylation status were analyzed through the counterfactual approach^26,27^ by decomposing the total effect of PM_2.5_ (and NO_x_) on dementia into 4 potential causal pathways: (1) a direct effect (pathways associating air pollution and dementia independently of serum markers of methylation status); (2) the effect due to mediation alone (pathways associating air pollution and dementia only through serum markers induced by air pollution); (3) the effect of interaction between air pollution and serum markers (pathways that only operate when both increased serum biomarkers and air pollution are present without mediation effect); and (4) the effect due to both mediation and interaction (pathways that only operate when both increased serum biomarkers and air pollution are present with mediation effect). The decomposition allows estimating and testing the proportion of the total effect due to each of these 4 components. The 4-way decomposition of the total effect requires jointly testing the associations between the exposures and the mediators/modifiers, which were assessed using linear or logistic regression, and the associations between the exposures and outcome, which were assessed using survival model. eFigure 1 (links.lww.com/WNL/C980) shows the 4 different pathway models.

To test whether CVDs played a role in the associations, we repeated the analyses after excluding those who developed CVDs during the follow-up (cases with incident CVD: 364).

Potential confounding factors were a priori identified–based literature review^2^ and available data from the study population: age, sex, educational level, year of assessment, smoking, early retirement, socioeconomic position, physical activity, creatinine, and use of supplements.

#### Secondary Analyses

To evaluate the potential modifier effects of sex and APOE, we tested for interaction between air pollutants and both sex and *APOE* genotype and reported the analyses by sex and *APOEε4* carriers/noncarriers. As sensitivity analysis, we additionally adjusted the models for food intake of folate and vitamin B_12_. All statistical analyses were performed with Stata, version 17 (StataCorp, TX).

### Data Availability

Access to the data for this study (snac-k.se) will be possible on request and approval by the SNAC-K data management and maintenance committee at the Aging Research Center, Karolinska Institutet, Stockholm, Sweden.

## Results

The analytical sample resulted in 2,512 dementia-free individuals because we excluded 240 persons affected by dementia at baseline, 1 person with intellectual disability, and 4 participants with tHcy levels >69.97 μmol/L or creatinine levels >400 μmol/L. In addition, 410 individuals had missing information on biomarkers or air pollution, and 196 had missing dementia information at follow-up. Those with missing information on exposure or covariates were more likely to be older (*p* < 0.001), while no differences concerning sex and education arose (*p* = 0.865 and *p* = 0.920, respectively).

During an average period of 5 years (mean: 5.18; SD: 2.96 years; range 2.1–10.3 years), 376 cases with incident dementia (incidence rate per 1,000 persons/year: 28.9; 95% CI 26.1–31.9) were identified. Baseline characteristics of the analytic sample overall and by incident dementia are reported in Table 1. Participants who developed dementia were less educated, less likely to be male, more likely to be/have been blue collar, and retired before the age of 65 years. Higher tHcy and lower methionine concentrations were observed among participants who developed dementia compared with those who did not.

**Table 1:**
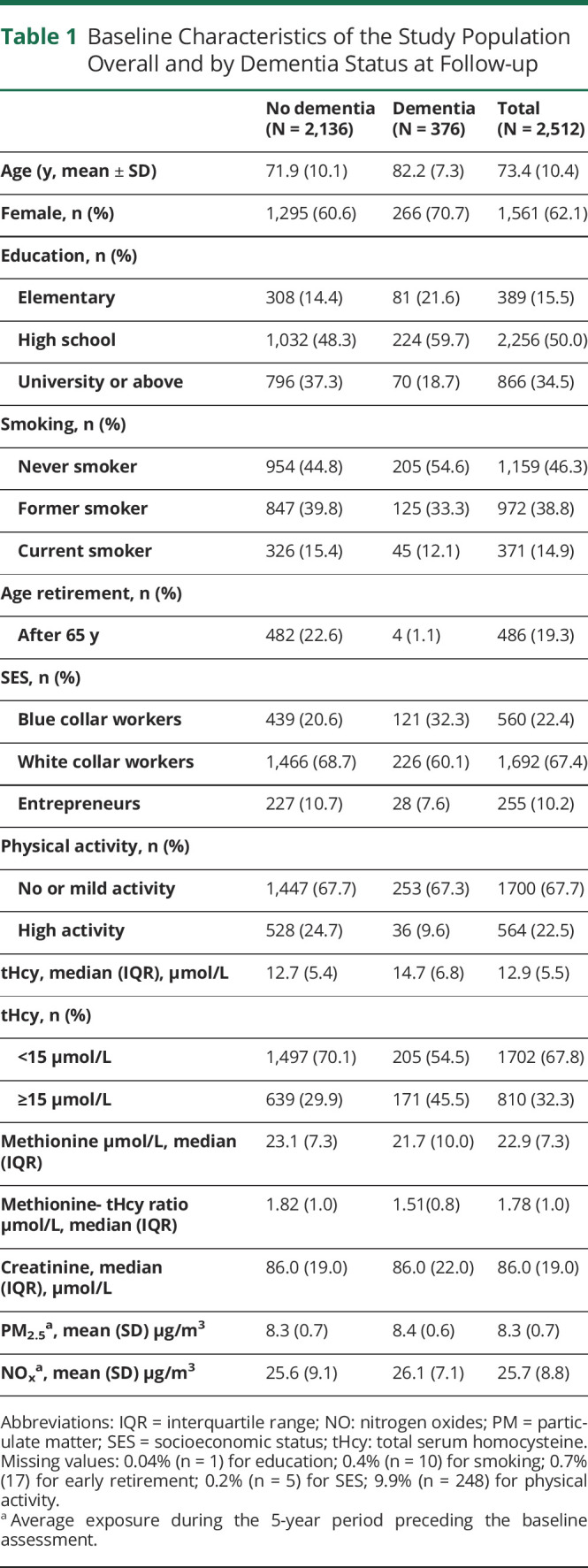
Baseline Characteristics of the Study Population Overall and by Dementia Status at Follow-up

Figure 1 shows the average concentration (µg/m^3^) of PM_2.5_ and NO_x_ during the 5 years preceding baseline assessment. During the 5 years before baseline, there was a slight decrease in the concentration of both air pollutants, specifically −2.04 µg/m^3^ (SD: 0.02) for PM_2.5_ and −4.68 µg/m^3^ (SD: 2.03) for NO_x_.

**Figure:**
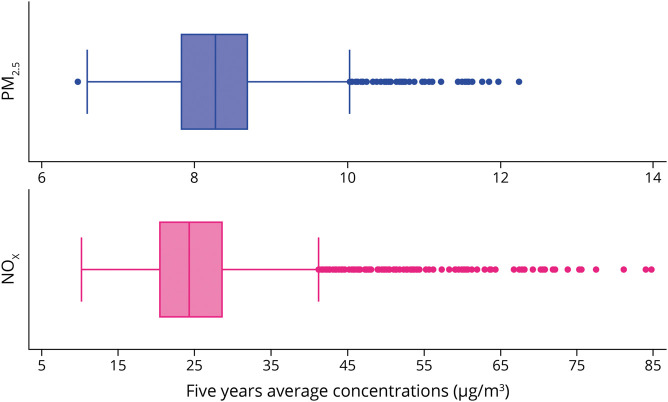
Concentration Levels of PM_2.5_ and NO_x_ During the 5 Years Preceding Baseline Assessment

A higher 5-year mean residential outdoor PM_2.5_ concentration was associated with higher tHcy and methionine levels at baseline, by 0.36 µmol/L for tHcy (95% CI 0.07–0.66) and 0.36 µmol/L (95% CI 0.02–0.70) for methionine, per 1 µg/m^3^ PM_2.5_, after adjusting for potential confounders (age, sex, education, smoking, retirement status, socioeconomic position, physical activity, creatinine level, and use of supplements). Similarly, higher levels of NO_x_ were associated with a higher tHcy concentration, by 0.31 (95% CI 0.07–0.5) per 10 µg/m^3^ increase in 5-year mean NO_x_. NO_x_ levels were not associated with a difference in methionine (mean difference µmol/L:0.15; 95% CI -0.13; 0.42 per 10 µg/m^3^ increase in NO_x_). Neither PM_2.5_ nor NO_x_ concentrations showed a significant association with Met:tHcy.

In adjusted models, a tHcy concentration above 15 µmol/L was associated with 55% higher hazard of dementia (HR: 1.55; 95% CI 1.23–1.95), and methionine above 20.7 µmol/L was associated with approximately 30% lower hazard to develop dementia (HR: 0.69; 95% CI 0.56–0.85). Met:tHcy ratio above 1.47 µmol/L was associated with 37% lower hazard of developing dementia (HR: 0.63; 95% CI 0.51–0.79).

After considering potential confounders, 70% higher incidence of dementia was found per 1 µg/m^3^ increase of PM_2.5_ (HR: 1.71; 95% CI 1.33–2.09), whereas a 30% increased risk of dementia was found per 10 µg/m^3^ increase of NO_x_ (HR: 1.33; 95% CI 1.19–1.49).

Table 2 summarizes the association between PM_2.5_ and incident dementia decomposed by tHcy and methionine. We found that 51.6% of the total effect of PM_2.5_ on dementia was due to mediation and/or interaction with tHcy. Overall, 47.8% of the association was explained only by interaction, while 6.6% only by mediation. Furthermore, 48.4% of the association between PM_2.5_ and dementia could be attributed to a direct effect.

**Table 2:**
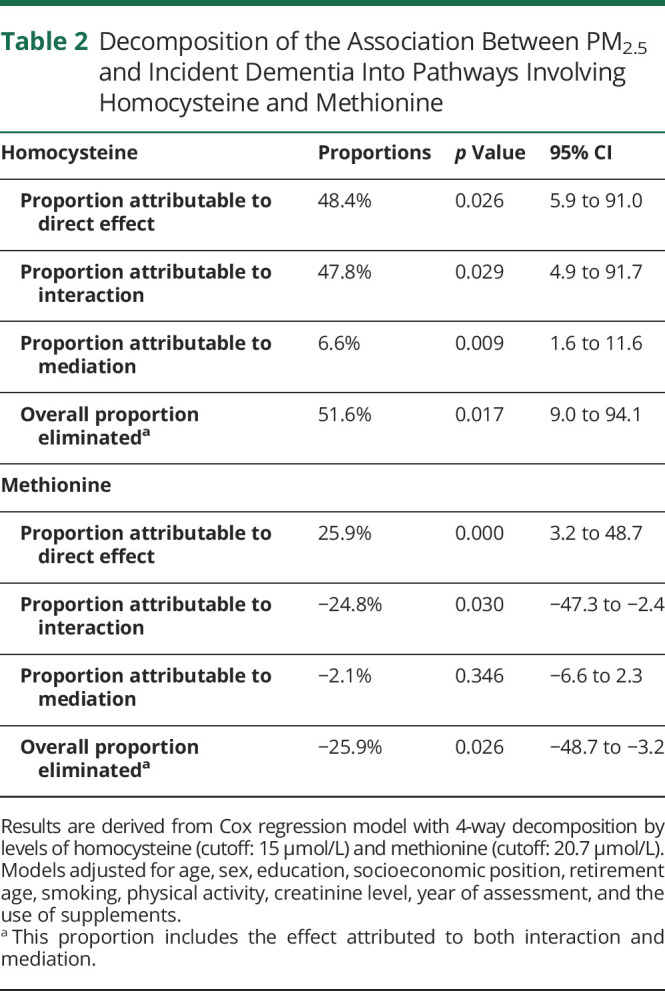
Decomposition of the Association Between PM_2.5_ and Incident Dementia Into Pathways Involving Homocysteine and Methionine

High concentrations of methionine reduced dementia risk linked to PM_2.5_ exposure by 26%. No statistically significant mediation effect was found through methionine in the association between PM_2.5_ and dementia. A direct effect of PM_2.5_ on dementia was also found here.

Similar, but attenuated, results were obtained for NO_x_ (eTable 2, links.lww.com/WNL/C980).

eTable3 (links.lww.com/WNL/C980) summarizes the association between PM_2.5_ and NO_x_ and incident dementia decomposed by Met:tHcy. A high Met:tHcy ratio was associated with a reduced dementia risk (−44.5%; 95% CI −83.0 to −5.9) per 1 µg/m^3^ increase of PM_2.5_. This was mainly due to a large proportion attributable to interaction between PM_2.5_ and Met:tHcy, while no mediation effect was detected. Similar results were obtained for NO_x_.

The results for tHcy were attenuated and no longer statistically significant, whereas the results remained similar for methionine when we repeated the analyses after excluding individuals with incident CVDs (Table 3). For PM_2.5_, neither the mediating nor the interaction roles were significant, while a direct effect was present (65.6%; 95% CI 6.4–100). High concentrations of methionine reduced the dementia risk linked to PM_2.5_ exposure by 30%, but mediation remained nonsignificant. Similar results were obtained for NO_x_ (eTable 4, links.lww.com/WNL/C980).

**Table 3:**
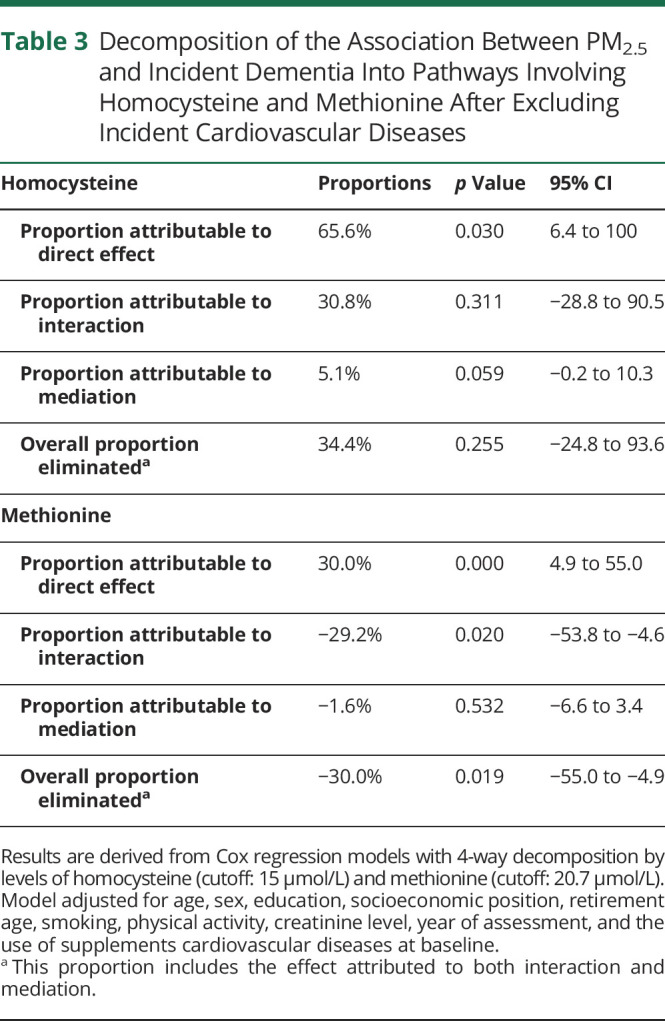
Decomposition of the Association Between PM_2.5_ and Incident Dementia Into Pathways Involving Homocysteine and Methionine After Excluding Incident Cardiovascular Diseases

### Secondary Analyses

We tested the modifying effect of sex and *APOE* genotype and reported the results in eTable 5 (links.lww.com/WNL/C980). Overall, 1 µg/m^3^ increase of PM_2.5_ was associated with an 87% (1.61–2.16) higher hazard for dementia in women and a 66% (1.32–2.09) increased hazard in men, while a 10 µg/m^3^ increase of NO_x_ was linked with a 3% increased dementia risk both in women (1.02–1.04) and men (1.01–1.05). Concerning *APOE* genotype, 1 µg/m^3^ increase of PM_2.5_ was associated with a 78% (1.53–2.0) higher hazard for dementia in APOEε4 noncarriers and an 83% (1.48–2.26) increased hazard in ε4 carriers, while a 10 µg/m^3^ increase of NO_x_ was linked with a 3% increased dementia risk both in noncarriers (1.02–1.04) and carriers (1.01–1.05). When we further adjusted the analyses for food intake of folate and vitamin B_12_, the results were consistent for homocysteine while slightly attenuated for methionine (eTable 6, links.lww.com/WNL/C980).

## Discussion

This study led to the following main findings: First, similar to previous studies, air pollution emerged as a risk factor of dementia development; Second, homocysteine and methionine, and the Met:tHcy ratio (an indirect sign of methylation activity), played a relevant role in this association; Third, their role was mainly as effect modifiers, with homocysteine exacerbating, while methionine mitigating the effect; Finally, the role played by homocysteine was observed only in participants who had developed CVDs, whereas the protective role of methionine was likely independent of CVD development.

Air pollution has recently been included in the list of modifiable risk factors of dementia.^1^ Despite methodological heterogeneity in study designs, differences in exposure assessments, and different settings, most recent studies provide evidence in support of an association, especially when considering PM_2.5_ exposure.^2^ In our study, we observed a 70% and 30% increased dementia risk per 1 µg/m^3^ and 10 µg/m^3^ increase in PM_2.5_ and NO_x_, respectively. These results come from an urban area in central Stockholm where substantial improvements in air quality have occurred in the past decade and where mean concentrations of air pollutants are quite low in comparison with the average in the rest of Europe, United States, or China. As a comparison with our mean concentration of 8.3 μg/m^3^, a recent study^28^ in Europe reported an average annual concentration of PM_2.5_ in 2019 of 13.8 μg/m^3^, but it is noteworthy that mean concentrations in our study were higher compared with those of 5.7 μg/m^3^ reported in the Nordic countries. Our results are in line with other Swedish studies investigating air pollution and dementia onset^29,30^ and suggest once again that even low concentrations of air pollution are associated with poor health outcomes.^31,32^ However, unlike previous studies that have reported stronger associations between PM_2.5_ and dementia in women when compared with men,^33^ we were unable to detect such a difference. Identifying vulnerable subgroups who might particularly benefit of air quality improvements continues to be important to safeguard public health and in setting efficient and appropriate air quality standards.^34^

In this study, we found that higher concentrations of tHcy increased the detrimental effect of air pollution by 50%, whereas raised methionine reduced the air pollutant–related dementia risk by 30%. We also found that a higher Met:tHcy ratio was associated with a reduced dementia risk related to air pollution exposure. Met:tHcy ratio can be considered a possible indicator of methylation activity, and reduced values of this ratio reflect impaired methylation activity. Hcy reflects the functional status of 3 B vitamins (folate, vitamin B_12_, and vitamin B_6_),^13^ and a number of factors can, directly or indirectly, raise blood concentration of tHcy, including age, renal impairment, and B vitamin insufficiency.^10^ Notably, it has been suggested that PM exposure may raise Hcy levels by inducing systemic inflammation and oxidative stress, reducing the activity of enzymes implicated in Hcy metabolism and/or competing with methyl groups with the Hcy remethylation process.^17^

According to our findings, for both homocysteine and methionine, interaction with air pollution seemed to be stronger than mediation. Our findings on interaction demonstrate an interplay between hyperhomocysteinemia and low methionine levels with air pollution determining the individual's dementia risk. This is consistent with the fact that hyperhomocysteinemia and low levels of methionine can be generated through pathways other than high air pollution exposure, as mentioned earlier.^10^

The mechanisms by which air pollution affects brain health are still mostly unknown. A study including transgenic mice exposed to urban nanosized PM showed increased cerebral Aβ deposition.^5^ This evidence was corroborated by a study from the US including 18,000 cognitively impaired individuals where^6^ the authors found that exposure to PM_2.5_ was associated with deposition of brain Aβ plaques. According to our findings, a direct effect of air pollution on dementia was responsible for up to 46.7% of the total effect, suggesting that alternative pathways may be at place, among which it can be hypothesized a deposition of amyloid plaques. Air pollution can also affect the brain through indirect pathways; for example, a close heart-brain connection is at play in dementia development,^35^ and it is well established that different components of air pollution increase cardiovascular morbidity and mortality.^36,37^ In line with these observations, we and others previously found that the presence of CVDs enhanced the detrimental effect of air pollution,^8^ and, particularly, stroke was an important intermediate condition between air pollution and dementia.^7^ Current knowledge supports 2 main pathways, namely a direct damage of air pollution to the brain and indirect pathways, including the development of CVDs. It is plausible to hypothesize that these pathways act in synergy being more complementary than alternative in affecting brain health.

In our study, we did not find any relevant mediation/interaction through tHcy when we excluded individuals who developed CVDs, and this sheds light on the possible mechanisms through which tHcy acts in this association, adding weight to the crucial role played here by CVDs. In an autopsy study investigating the impact of tHcy on different neuropathologic outcomes, the relationship between tHcy and neurofibrillary tangles was limited to individuals who also had cerebral infarcts, suggesting that cerebral perfusion may modulate the impact of homocysteine on tau pathology.^38^ In our study, after the exclusion of cases with incident CVD, the proportion attributable to the direct effect of pollution rose to 65% of the total effect, thus suggesting that air pollution acts through multiple pathways including direct effects without the involvement of tHcy in addition to indirect effects through CVDs partly influenced by tHcy.

An attenuation of the effect after excluding cases with CVD was not detectable for methionine, suggesting that its protective role could act through pathways other than reduced cardiovascular burden. Methionine may in fact have a more pleiotropic role than tHcy, being potentially involved in processes not primarily linked with CVDs, but eventually affecting brain aging. Indeed, methionine is involved in processes such as protein synthesis and polyamine metabolism and serves as the precursor to produce amino acids involved in normal brain function.^39,40^ Methionine deficiency has been linked with glutathione deficiency, a major antioxidant, associated with the development of several diseases.^41^ Interventions that lower tHcy and increase methionine, such as improving B vitamin status,^13^ might thus modify the association between air pollution and dementia.

Our findings are based on a large, clinically characterized, population-based study with spatially detailed information on long-term exposure to air pollution, biomarkers, and clinical evaluations including dementia diagnosis. Some limitations need to be acknowledged. In the SNAC-K study, we lack a biological characterization of dementia subtypes, and further studies are needed to better understand whether air pollution raises specific subtype risk (e.g., AD, vascular dementia, and Lewy body dementia). We, however, would like to point out that in the SNAC-K study, most of the cases with dementia are late onset, and previous studies have shown that dementia occurring after the age of 75 years is rarely purely AD but more commonly demonstrating a mixed pathology.^42^ In addition, the geographical area included, the Kungsholmen district of Stockholm, is small and limits spatial contrasts in air pollutants. Furthermore, the SNAC-K study includes older adults who are generally wealthy, fit, and healthy and live in an area with relatively low air pollution levels, which might limit the generalizability of our findings to other populations. However, this would most likely lead to a possible underestimation rather than inflation of the association between air pollution and dementia and coupled with the observations of harmful effects at low exposure levels; this strengthens the clinical and public health message. In this study, air pollutant exposure was assessed since 1990 and is assumed to temporally overlap with the long preclinical and prodromal stages of dementia during which lesion deposition and accumulation can occur. Future studies challenging that assumption with even longer observational period should be undertaken, but irrespectively, our findings likely support the role of air pollution as an exacerbation of dementia development. We were limited to using serum biomarkers from a single time point, and to confirm our findings, further evaluations on persistent levels of high homocysteine and low methionine during follow-up time are needed. In addition, even if we were able to adjust the analyses for several covariates, including use of supplements and for food intake of folate and vitamin B_12_, we did not have available data on the frequency and dosage of supplements. Finally, alternative or complementary biological pathways might be at play in the air pollution/dementia link, for example, inflammation, and these hypotheses should be tested in future studies.

Collectively, our findings support the evidence of air pollution as a risk factor of dementia in older adults. This was evident in an area with comparatively high standard of air quality and argues for additional actions in further reducing air pollution in our cities. We here found, once again, that an indirect link between air pollution and dementia may exist and that the homocysteine/methionine cycle and cardiovascular burden may be implicated. In addition to these pathways, our results indicated a substantial direct effect of air pollution on dementia, suggesting that air pollution affects the development of dementia through multiple pathways. This highlights the need to further elucidate the exact biological mechanisms behind the brain damage of air pollution.

## Glossary

APOE: Apolipoprotein E
CVD: cardiovascular disease
HR: hazard ratio
tHcy: homocysteine
